# Leptin sensitizing effect of 1,3-butanediol and its potential mechanism

**DOI:** 10.1038/s41598-021-96460-y

**Published:** 2021-09-06

**Authors:** Masayo Isoda, Ken Ebihara, Nagisa Sawayama, Akiko Murakami, Chihiro Ebihara, Koji Shibuya, Akihito Takei, Shoko Takei, Tetsuji Wakabayashi, Daisuke Yamamuro, Manabu Takahashi, Shuichi Nagashima, Shun Ishibashi

**Affiliations:** Division of Endocrinology and Metabolism, Department of Internal Medicine, Jichi Medical University School of Medicine, Yakushiji 3311-1, Shimotsuke, Tochigi 329-0498 Japan

**Keywords:** Endocrine system and metabolic diseases, Obesity

## Abstract

Leptin is an adipocyte-derived hormone that regulates appetite and energy expenditure via the hypothalamus. Since the majority of obese subjects are leptin resistant, leptin sensitizers, rather than leptin itself, are expected to be anti-obesity drugs. Endoplasmic reticulum (ER) stress in the hypothalamus plays a key role in the pathogenesis of leptin resistance. ATP-deficient cells are vulnerable to ER stress and ATP treatment protects cells against ER stress. Thus, we investigated the therapeutic effects of oral 1,3-butanediol (BD) administration, which increases plasma β-hydroxybutyrate and hypothalamic ATP concentrations, in diet induced obese (DIO) mice with leptin resistance. BD treatment effectively decreased food intake and body weight in DIO mice. In contrast, BD treatment had no effect in leptin deficient *ob/ob* mice. Co-administration experiment demonstrated that BD treatment sensitizes leptin action in both DIO and *ob/ob* mice. We also demonstrated that BD treatment attenuates ER stress and leptin resistance at the hypothalamus level. This is the first report to confirm the leptin sensitizing effect of BD treatment in leptin resistant DIO mice. The present study provides collateral evidence suggesting that the effect of BD treatment is mediated by the elevation of hypothalamic ATP concentration. Ketone bodies and hypothalamic ATP are the potential target for the treatment of obesity and its complications.

## Introduction

Leptin is an adipocyte-derived hormone that regulates energy homeostasis mainly via the hypothalamus^1–3^. Leptin enters the brain from the circulatory system through the blood–brain barrier^4^ and binds to receptors expressed in the hypothalamus, particularly in the arcuate nucleus^5^, and reduces food intake and body weight^6^. Circulating leptin level increases in proportion to body fat content^7^. Although most obese subjects have high serum leptin levels, increased leptin fails to suppress the progression of obesity. Indeed, it has been demonstrated that obese humans are weakly responsive to exogenously administered leptin in terms of body weight reduction^8^. This pathological condition is called leptin resistance. Today, leptin sensitizers rather than leptin itself are expected to be anti-obesity drugs.

Although the precise mechanism underlying leptin resistance has not been elucidated, it has been reported that endoplasmic reticulum (ER) stress in the hypothalamus plays a key role in the pathogenesis of leptin resistance^9,10^. It has also been demonstrated that chemical chaperones such as 4-phenyl butyric acid and tauroursodeoxycholic acid, which have the ability to decrease ER stress, act as leptin-sensitizing agents^10^. Under ER stress, the accumulation of misfolded proteins facilitates the unfolded protein response (UPR) which requires appreciable amounts of ATP^11,12^. Indeed, ATP-deficient cells are vulnerable to ER stress, and ATP treatment protects cells against ER stress^13^. For these reasons, increased hypothalamic ATP concentration might have a protective effect against ER stress and leptin resistance in the hypothalamus.

Ketone bodies have been demonstrated to increase ATP concentrations in the central nervous system^14–16^. A simple way to increase the circulating concentration of ketone bodies is through a ketogenic diet^17^. Ketogenic diet is a low carbohydrate and high fat diet. Lack of carbohydrate induces the conversion of fat to fatty acids and ketone bodies in the liver. However, the effects of ketogenic diets on body weight and food intake are contradictory^18,19^. Ketosis induced by ketogenic diet has been reported to act both orexigenically and anorexigenically in animal studies^18,19^. One of the reasons why the ketogenic diet has two conflicting actions is that it is both ketogenic and low carbohydrate. Low carbohydrate diet can act similarly to calorie restriction, increasing food intake^20,21^. On the other hand, ketone bodies have been demonstrated to increase ATP concentrations in the central nervous system^14–16^. If the elevation of hypothalamic ATP concentration has a protective effect against ER stress and leptin resistance in the hypothalamus, then ketone bodies should reduce food intake and body weight in obese subjects with leptin resistance. However, the effects of ketone bodies on hypothalamic ER stress and leptin resistance have not been reported.

The aim of this study is to investigate the effect of ketone bodies on the leptin sensitivity in mice. 1,3-butanediol (BD), a precursor of β-hydroxybutyrate (βHB), was used to increase plasma ketone body concentrations. BD is an ethanol dimer (CH_3_–CHOH–CH_2_–CH_2_OH), one of four stable structural isomers of butanediol^22^. It is converted to βHB in the liver^23–25^. Oral BD administration has been demonstrated to increase plasma βHB concentration^26,27^. In addition, it was also demonstrated that oral administration of esterified BD reduces body weight in diet induced obese (DIO) mice although the involvement of leptin sensitization was not investigated^28^. In this study, instead of mixing BD into a diet, BD was administered to mice by oral gavage to avoid the change of the diet composition. BD treatment effectively reduced food intake and body weight in diet induced obese (DIO) mice which were leptin resistant. In contrast, no effect of BD treatment was observed in leptin deficient *ob/ob* mice. Co-administration experiment of BD and leptin demonstrated that BD treatment sensitizes leptin action in both DIO and *ob/ob* mice. These results indicate that leptin is necessary for BD treatment to exert its effects, and that leptin sensitization is the primary effect of BD treatment on body weight. It was also confirmed that BD treatment attenuated ER stress and leptin resistance at the hypothalamic level in DIO mice. This is the first report demonstrating the leptin sensitizing effect of BD treatment in DIO mice with leptin resistance. The present study suggests the possibility that the effect of BD treatment is mediated by the elevation of hypothalamic ATP concentration. Ketone bodies and hypothalamic ATP are potential targets for the treatment of obesity and its complications.

## Results

### Therapeutic effect of BD treatment on DIO mice

**Figure 1:**
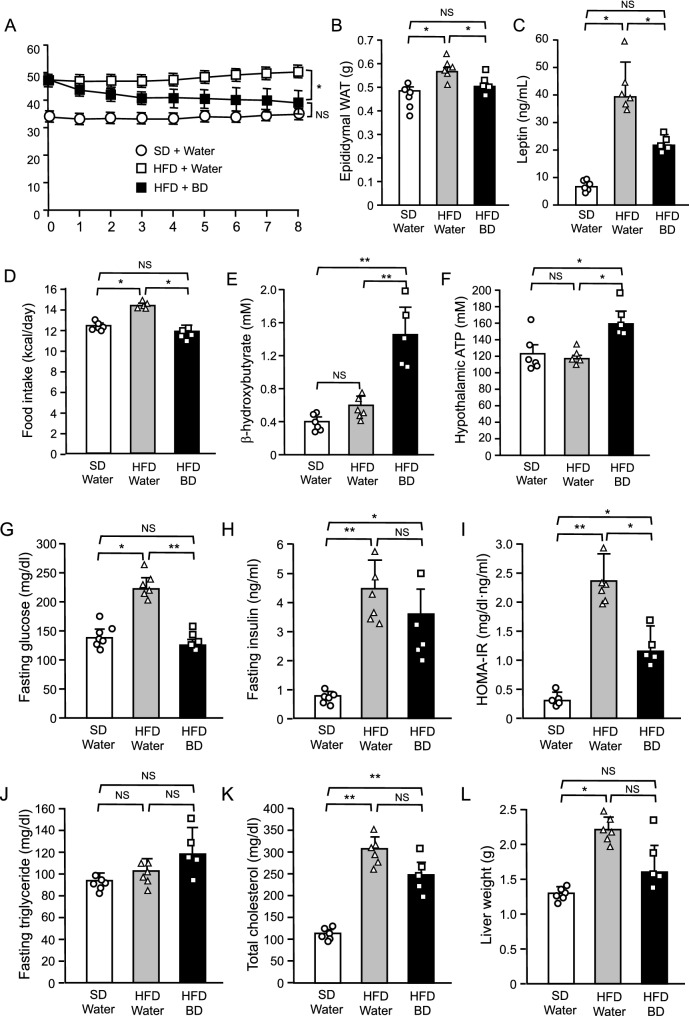
Therapeutic effect of oral BD administration on DIO mice. (**A**) Body weight change, (**B**) epididymal WAT weight, (**C**) plasma leptin concentrations, (**D**) food intake, (**E**–**H**) plasma β-hydroxybutyrate, hypothalamic ATP, fasting plasma glucose and fasting plasma insulin concentrations, (**I**) HOMA-IR, (**J**,**K**) fasting plasma triglyceride and plasma total cholesterol concentrations and (**L**) liver weight in mice treated with water or BD under SD or HFD. Values are means ± SEM (*n* = 6 for SD + Water, 6 for HFD + Water, 5 for HFD + BD). NS, not significant, *P < 0.05 (Bonferroni's test) (**A**). NS, not significant, *P < 0.05, **P < 0.01 (Student’s *t*-test) (**B**–**L**).

To investigate the therapeutic effect of ketone bodies on DIO, we administered BD or water orally to DIO mice twice a day for 8 weeks under HFD. As a control, we administered water to lean mice twice a day for 8 weeks under SD. Body weight in DIO mice was effectively decreased by BD treatment and was not significantly different from that in lean control mice fed SD at the end of the experiment (Fig. 1A). Consistent with the body weight change, BD treatment decreased epididymal WAT weight in DIO mice to the same level as in lean control mice (Fig. 1B). We measured plasma leptin concentrations as an indicator of total body fat mass. In DIO mice, plasma leptin concentrations were markedly elevated and were significantly reduced by BD treatment (Fig. 1C). The elevated daily food intake in DIO mice was also decreased by BD treatment to the same level as in lean control mice fed SD (Fig. 1D). We measured plasma β-hydroxybutyrate and hypothalamic ATP concentrations at the end of the experiment. Plasma β-hydroxybutyrate concentrations in DIO mice treated with water tended to be higher than in lean control mice but there was no statistically significant difference (Fig. 1E). BD treatment effectively increased plasma β-hydroxybutyrate concentrations in DIO mice. Consistent with these plasma β-hydroxybutyrate concentrations, there was no difference in hypothalamic ATP concentrations between lean control and DIO mice treated with water and BD treatment significantly increased hypothalamic ATP concentrations in DIO mice (Fig. 1F).

Next, we investigated the effect of BD treatment on glucose and lipid metabolisms in DIO. In DIO mice, fasting plasma glucose and insulin concentrations and HOMA-IR were apparently elevated when compared with lean control mice (Fig. 1G–I). However, BD treatment decreased fasting glucose concentrations in DIO mice to the same level as in lean control mice. Although fasting insulin concentrations in DIO mice were not significantly decreased, HOMA-IR in DIO mice was significantly decreased by BD treatment. As to lipid metabolism, BD treatment had no effect on fasting plasma triglyceride and total cholesterol concentrations but showed a tendency to decrease the liver weight in DIO mice (Fig. 1J–L).

### Effect of BD treatment in lean control mice

**Figure 2:**
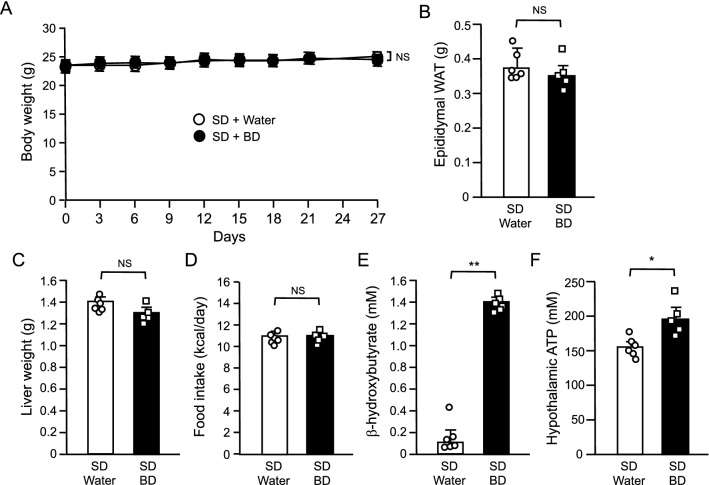
Effect of oral BD administration in WT mice under SD. (**A**) Body weight change, (**B**) epididymal WAT weight, (**C**) liver weight, (**D**) food intake, (**E**) plasma β-hydroxybutyrate and (**F**) hypothalamic ATP concentrations. Values are means ± SEM (*n* = 6 for SD + Water, 5 for SD + BD). NS, not significant (Bonferroni's test) (**A**). NS, not significant, *P < 0.05, **P < 0.01 (Student’s *t*-test) (**B**–**F**).

We also investigated the effect of oral BD administration in lean control mice under SD. Twice daily oral administration at the same dose (5 g/kg) used in DIO mice had any apparent effect on neither body weight nor tissue weights including epididymal WAT and liver in lean control mice (Fig. 2A–C). BD treatment also had no effect on food intake while both plasma β-hydroxybutyrate and hypothalamic ATP concentrations were significantly elevated by BD treatment (Fig. 2D–F).

### Effect of BD treatment in *ob/ob* mice with or without leptin treatment

**Figure 3:**
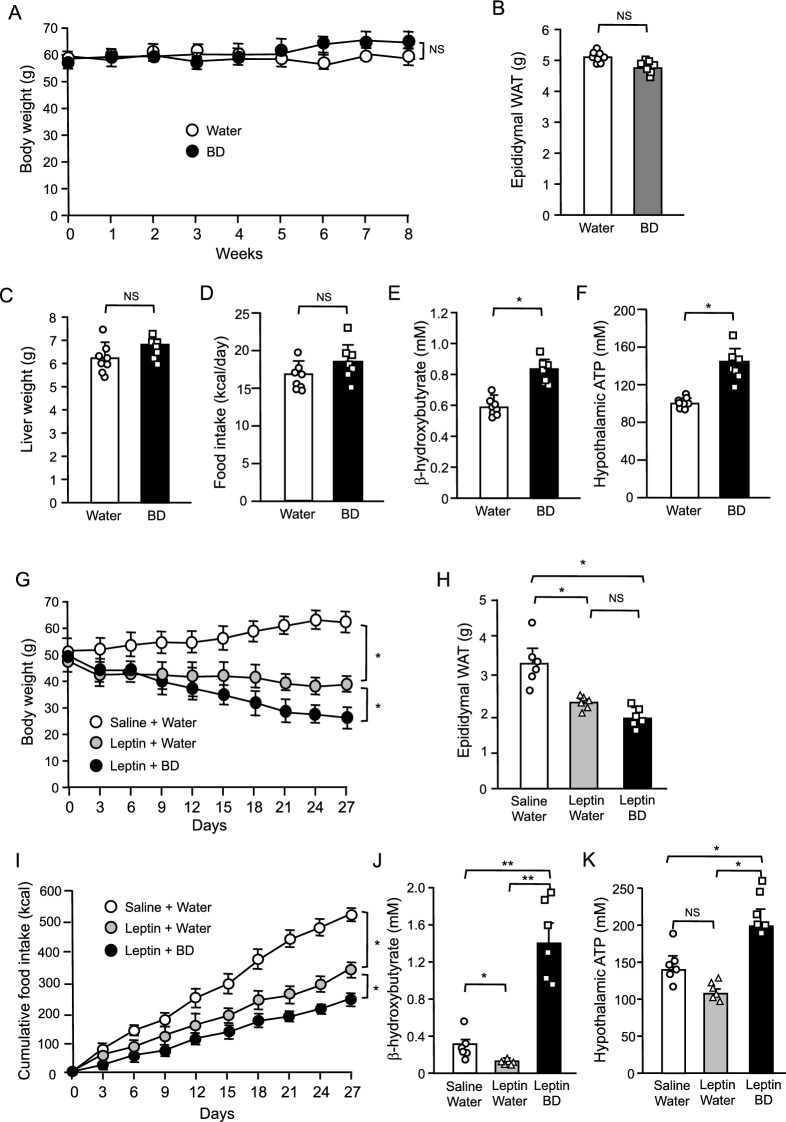
Effects of oral BD administration (**A**–**F**) and co-administration of oral BD and intraperitoneal leptin (**G**–**J**) in *ob/ob* mice. (**A**) Body weight change, (**B**) epididymal WAT weight, (**C**) liver weight, (**D**) food intake, (**E**) plasma β-hydroxybutyrate and (**F**) hypothalamic ATP concentrations in BD single administration experiment. (**G**) Body weight change, (**H**) epididymal WAT weight, (**I**) cumulative food intake, (**J**) plasma β-hydroxybutyrate and (**K**) hypothalamic ATP concentrations in leptin and BD co-administration experiment. Values are means ± SEM (**A**–**F**, *n* = 8 for Water, 7 for BD; **G**–**K**, *n* = 6 for Saline + Water, 6 for Leptin + Water, 6 for Leptin + BD). NS, not significant, *P < 0.05 (Bonferroni's test) (**A**,**G**,**I**). NS, not significant, *P < 0.05, **P < 0.01 (Student’s *t*-test) (**B**–**F**, **H**,**J**,**K**).

BD treatment did not significantly reduce the body weight in lean control mice at the same dose (5 g/kg) that was effective in DIO mice (Figs. 1A, 2A). Lean mice had very low levels of circulating leptin when compared with DIO mice (Fig. 1C), suggesting that the therapeutic effect of BD treatment requires a decent amount of leptin. To investigate the role of leptin in the effect of BD treatment, we examined the effect of BD treatment in *ob/ob* mice which genetically lack circulating leptin^1^. Consistent with our expectations, BD treatment did not significantly reduce the body weight in *ob/ob* mice at the dose (5 g/kg) that were effective in DIO mice (Fig. 3A). In addition, epididymal WAT and liver weights (Fig. 3B,C) and food intake (Fig. 3D) in these mice were unchanged by BD treatment. At this time, we confirmed the significant elevation of both plasma β-hydroxybutyrate and hypothalamic ATP concentrations by BD treatment in *ob/ob* mice (Fig. 3D,E).

We could not deny the possibility that the dose and the administration used in this study were inadequate for *ob/ob* mice. Therefore, again we tested the effect of twice daily oral BD administration at the dose of 5 g/kg in *ob/ob* mice with leptin treatment. Once daily intraperitoneal leptin injection at the dose of 150 μg/kg effectively decreased body weight, epididymal WAT weight and food intake in *ob/ob* mice (Fig. 3G–I). Co-administration of BD further decreased body weight, epididymal WAT weight and food intake although the additional reduction of epididymal WAT weight was not statistically significant (Fig. 3G–I)). Leptin treatment increased neither plasma β-hydroxybutyrate nor hypothalamic ATP concentrations while BD treatment significantly increased them in *ob/ob* mice (Fig. 3J,K). These results indicate that BD treatment requires leptin to exert its therapeutic effect and that leptin sensitization is the main effect of BD treatment on body weight.

### Leptin sensitizing effect of BD treatment in mice under HFD

**Figure 4:**
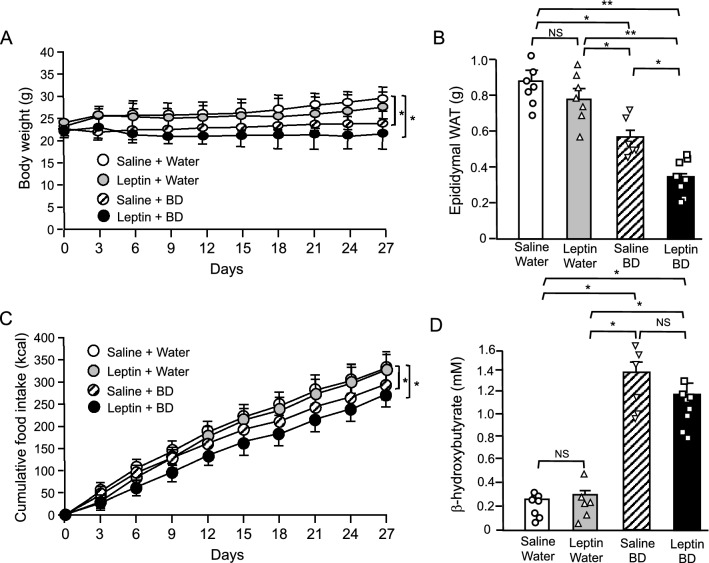
Effect of co-administration of oral BD and intraperitoneal leptin in WT mice under high fat diet. (**A**) Body weight change, (**B**) epididymal WAT weight, (**C**) cumulative food intake, (**D**) plasma β-hydroxybutyrate concentration. Values are means ± SEM (*n* = 7 for Saline + Water, 6 for Leptin + Water, 6 for Saline + BD, 9 for Leptin + BD). *P < 0.05, **P < 0.01 (Bonferroni's test) (**A**,**C**). NS, not significant, *P < 0.05, **P < 0.01 (Student’s *t*-test) (**B**,**D**).

To investigate the leptin sensitizing effect of BD treatment, we treated mice with leptin and/or BD under HFD. Under HFD, once daily leptin injection at the dose of 150 μg/kg had no significant effect on body weight, epididymal WAT weight and food intake (Fig. 4A–C), indicating that HFD induced leptin resistance in mice as reported previously^29,30^. On the other hand, BD treatment significantly decreased body weight, epididymal WAT weight and food intake and co-administration of leptin and BD further decreased them (Fig. 4A–C). At this time, we confirmed that BD treatment effectively increased plasma β-hydroxybutyrate concentrations irrespective of the presence or absence of leptin treatment (Fig. 4D). Leptin treatment had no effect on plasma β-hydroxybutyrate concentrations. Since leptin treatment alone had no significant effect on body weight, the effect of co-administration can be described as BD treatment sensitizes leptin action.

### Pair-feeding experiment in mice under HFD

**Figure 5:**
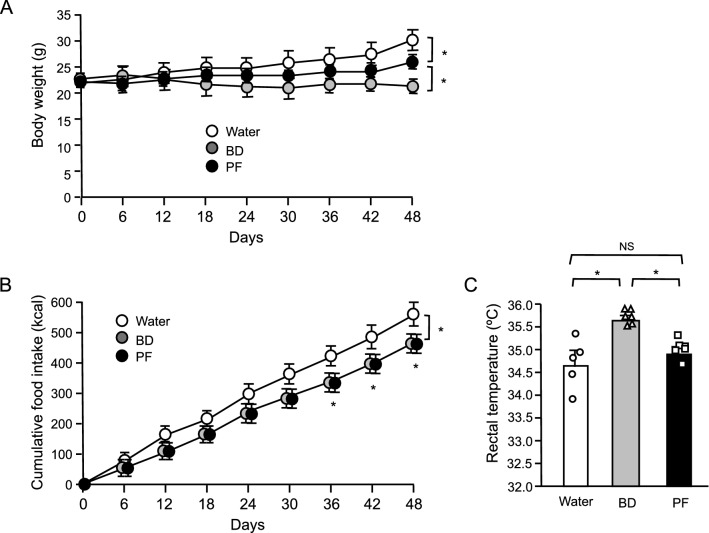
Effects of oral BD administration and pair-feeding (PF) in WT mice under high fat diet. (**A**) Body weight change, (**B**) cumulative food intake, (**C**) rectal temperature. Values are means ± SEM (*n* = 5 for Water, 6 for BD, 6 for PF). *P < 0.05 (Bonferroni's test) (**A**,**B**). NS, not significant, *P < 0.05 (Student’s *t*-test) (**C**).

If BD treatment sensitizes leptin action, BD treatment should not only suppress food intake but also increase energy expenditure. To evaluate the influence of food intake reduction by BD treatment on body weight, we performed the pair-feeding experiment in which mice were fed the same amount of food consumed by BD administered mice. Under HFD, BD treatment significantly decreased body weight and food intake (Fig. 5A,B). Pair-feeding also significantly decreased body weight (Fig. 5A). However, Body weight reduction by BD treatment was clearly greater than that by pair-feeding, suggesting that BD treatment induced both food intake reduction and energy expenditure. As expected, while pair-feeding had no effect on rectal temperature in mice, BD treatment significantly increased rectal temperature (Fig. 5C), indicating that BD treatment increased energy expenditure at least partly by thermogenesis.

### Effect of short-term BD treatment on leptin sensitivity in DIO mice

**Figure 6:**
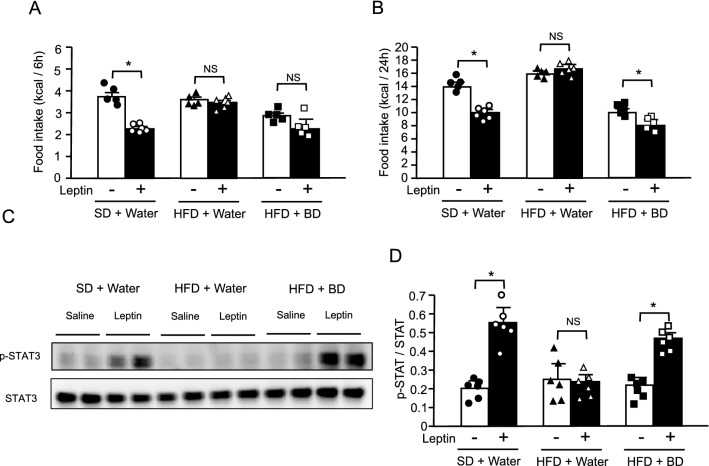
Effect of oral BD administration on leptin actions in DIO mice. (**A**) 6-h and (**B**) 24-h food intake after saline or leptin injection in lean control mice pretreated with water or DIO mice pretreated with water or BD. (**C**) Western blot analyses for p-STAT3 and total STAT3 protein levels 1 h after saline or leptin injection in the hypothalamus from lean control mice pretreated with water or DIO mice pretreated with water or BD. (**D**) Ratio of signal intensities of p-STAT3 to total STAT3 in C. Values are means ± SEM (*n* = 6 for each group). NS, not significant, *P < 0.05 (Student’s *t*-test).

To ensure that the leptin sensitizing effect by BD treatment is not secondary to body weight reduction or metabolic improvement, we examined the effect of short-term BD treatment in DIO mice. After 3-day administration of water or BD to DIO mice, saline or leptin was injected. Three-day BD treatment did not significantly change the body weight and blood glucose concentrations (data not shown). While leptin injection effectively decreased both 6-h and 24-h food intakes after injection in lean control mice, leptin injection had no effect on both 6-h and 24-h food intakes in DIO mice pretreated with water (Fig. 6A,B). However, leptin injection significantly decreased 24-h food intake in DIO mice pretreated with BD, although the reduction of 6-h food intake was not statistically significant. We also examined the leptin receptor signaling in the hypothalamus. Leptin injection effectively increased the phosphorylation of hypothalamic STAT3 in lean control mice but had no effect in DIO mice pretreated with water (Fig. 6C,D, Supplementary Fig. 6C). On the other hand, leptin injection significantly increased the phosphorylation of hypothalamic STAT3 in DIO mice pretreated with BD. These results demonstrate that short-term BD treatment sensitizes leptin action at the level of hypothalamus.

### Effect of BD treatment on ER stress in the hypothalamus of DIO and *ob/ob* mice

**Figure 7:**
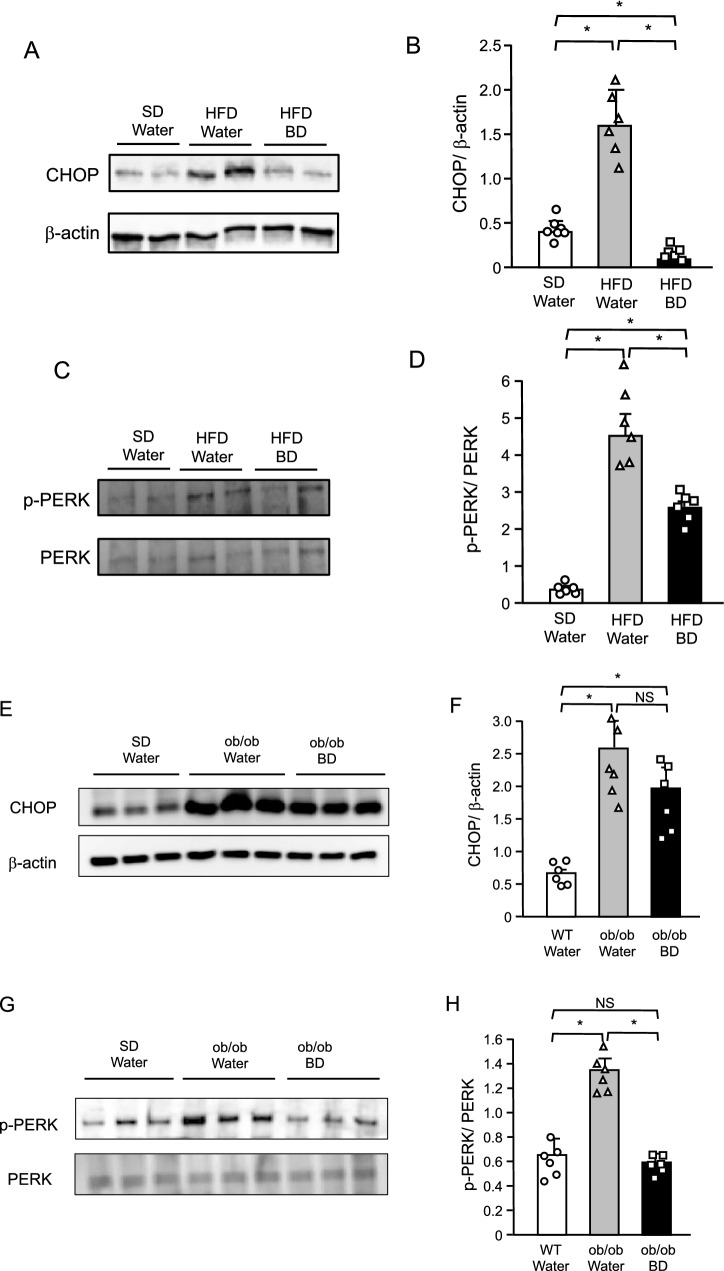
Effect of BD on expression of ER stress marker in the hypothalamus of DIO (**A**–**D**) and *ob/ob* (**E**–**H**) mice. (**A**,**E**) Western blot analyses for CHOP and β-actin protein levels in the hypothalamus. (**B**,**F**) Ratio of signal intensities of CHOP to β-actin in A and E. (**C**,**G**) Western blot analyses for p-PERK and total PERK protein levels in the hypothalamus. (**D**,**H**) Ratio of signal intensities of p-PERK to total PERK in C and G. Values are means ± SEM (**A**–**D**, *n* = 7 for SD + Water, 6 for HFD + Water, 6 for HFD + BD; **E**–**H**, *n* = 6 for WT + Water, 6 for *ob/ob* + Water, 6 for *ob/ob* + BD). NS, not significant, *P < 0.05 (Student’s *t*-test).

We investigated whether BD treatment attenuates ER stress in the hypothalamus of DIO mice. Water or BD were administered to DIO mice for 3 days. Expressions of ER stress markers, CHOP and the phosphorylation of PERK were markedly increased in DIO mice when compared with lean control mice (Fig. 7A–D, Supplementary Fig. 7A,C), indicating that HFD induced ER stress in the hypothalamus. However, short-term BD pretreatment effectively suppressed these increments of CHOP expression and PERK phosphorylation under HFD (Fig. 7A–D, Supplementary Fig. 7A,C), indicating that BD treatment attenuated ER stress in the hypothalamus under HFD. In this study, BD treatment sensitized leptin action in *ob/ob* mice (Fig. 3G–I). Therefore, we also examined expressions of ER stress markers in the hypothalamus sampled from *ob/ob* mice at the end of the chronic BD treatment experiment. Both expressions of CHOP and phosphorylated PERK were significantly increased in *ob/ob* mice treated with water when compared with lean control mice (Fig. 7E–H, Supplementary Fig. 7E,H), indicating that ER stress occurred in the hypothalamus of *ob/ob* mice. BD treatment did not significantly decreased CHOP expression but markedly decreased phosphorylation of PERK (Fig. 7E–H, Supplementary Fig. 7E,H), suggesting that BD treatment attenuated ER stress in the hypothalamus of *ob/ob* mice.

## Discussion

BD treatment effectively reduced body weight and food intake in DIO mice (Fig. 1A,D). Generally, DIO mice are made obese and leptin resistant with HFD loading^29,30^. DIO mice used in this study were also leptin resistant, as plasma leptin concentrations were markedly elevated (Fig. 1C). Thus, it can be said that BD treatment is effective for mice with leptin resistance. On the other hand, BD treatment had no effect in lean control mice fed SD (Fig. 2). Lean mice had very low circulating leptin levels than DIO mice (Fig. 1C), suggesting that the therapeutic effect of BD treatment requires a decent amount of leptin and/or leptin resistance. To investigate the role of leptin in the therapeutic effect of BD treatment, we treated genetically leptin deficient *ob/ob* mice with BD. As expected, BD treatment alone had no effect, but co-administration of BD with leptin resulted in greater weight loss in ob/ob mice than leptin treatment alone (Fig. 3). These results demonstrate that leptin is necessary for BD treatment to exert its effects, and that leptin sensitization is the primary effect of BD treatment on body weight.

We verified the leptin sensitizing effect of BD treatment under HFD feeding which induces leptin resistance. In mice fed HFD, while leptin treatment had no effect, co-administration of BD and leptin resulted in a significant decrease in body weight and food intake compared to BD treatment alone (Fig. 4). This certainly indicates that BD treatment sensitized leptin action. The decrease in body weight of leptin resistant mice after BD treatment alone is thought to be due to the sensitization of endogenous leptin by BD. Furthermore, pair-feeding experiment revealed that BD treatment decreased body weight by not only suppressed food intake, but also up-regulating energy expenditure (Fig. 5). This finding supports the idea that BD treatment sensitizes endogenous leptin, which is well known to suppress appetite and increase energy expenditure^31^.

To ensure that the leptin sensitizing effects by BD treatment are not secondary to body weight reduction or metabolic improvement, we examined the effects of short-term BD treatment in DIO mice. Although there were no significant changes in body weight or blood glucose levels after three days of BD treatment (data not shown), apparently sensitized leptin action in DIO mice in which exogenous leptin showed no effect (Fig. 6A,B). At this time, we examined the phosphorylation of STAT3 as a leptin receptor signal in the hypothalamus of DIO mice. Three-days BD treatment also increased leptin-induced phosphorylation of STAT3 (Fig. 6C,D). These results indicate that leptin sensitization by BD treatment is the primary effect at the hypothalamic level.

The aim of this study is to investigate the effect of ketone bodies on leptin sensitivity in mice. BD was used to raise the plasma ketone body concentration. We hypothesized that ketone bodies ameliorate ER stress and leptin resistance in the hypothalamus through the elevation of hypothalamic ATP concentration. The precise mechanism underlying leptin resistance has not been clarified. However, it has been reported that ER stress in the hypothalamus plays a key role in the pathogenesis of leptin resistance^9,10^. Under ER stress, the accumulation of misfolded proteins facilitates UPR, which requires appreciable amounts of ATP^11,12^. Thus, abundant ATP supply might have protective effect against ER stress. As expected, BD treatment decreased expressions of ER stress markers, which were elevated in the hypothalamus of DIO mice (Fig. 7A–D). These results support the view that the elevation of hypothalamic ATP concentration has a protective effect against ER stress.

Since BD treatment also sensitized leptin action in *ob/ob* mice (Fig. 3G–I), we examined the ER stress in the hypothalamus of *ob/ob* mice. Compared with lean control mice, ER stress markers were elevated in the hypothalamus of *ob/ob* mice (Fig. 7E–H), as reported previously^32^. BD treatment increased plasma βHB and hypothalamic ATP concentrations (Fig. 3D,E) and decreased expressions of ER stress markers in the hypothalamus in *ob/ob* mice (Fig. 7E–H). These results suggest that ketone bodies ameliorate ER stress and leptin resistance in the hypothalamus by increasing hypothalamic ATP concentration in not only DIO but also in *ob/ob* mice.

The effects of ketogenic diets on body weight and food intake have are contradictory^18^. One of the reasons for this contradiction is that ketogenic diet is high fat and low carbohydrate at the same time. To test the simple effect of ketone bodies on body weight and food intake, we used BD as a ketogenic supplement to increase plasma ketone body concentrations^26,27^. In addition, we administered BD to mice by oral gavage to avoid the change of the diet composition. BD administration by oral gavage effectively increased plasma β-hydroxybutyrate concentrations in mice. Thus, the present study could demonstrate the effect of ketone body itself on body weight and food intake.

We further investigated the effects of BD treatment on glucose and lipid metabolism in DIO. BD treatment significantly decreased fasting glucose levels and HOMA-IR (Fig. 1G,I), indicating that BD treatment improved insulin resistance in DIO mice. On the other hand, BD treatment caused little change in lipid biomarkers such as fasting plasma triglyceride and total cholesterol concentrations (Fig. 1J,K), although showed a tendency to decrease liver weight in DIO mice (Fig. 1L). At least, there were no adverse effects of BD treatment on lipid metabolism. The sick feelings or digestive problems caused by oral BD administration may have led to decreased food intake and body weight in DIO mice. If so, oral BD administration should decrease food intake and body weight in lean control and *ob/ob* mice. However, it had no effect on these mice. This means that oral BD administration did not cause any major digestive problems.

The present study provides collateral evidence that ketone bodies ameliorate ER stress and leptin resistance in the hypothalamus through the elevation of hypothalamic ATP concentration. However, although it has been reported that ATP treatment protects cultured cells against ER stress^13^, we did not directly prove that the elevation of ATP concentrations ameliorates ER stress in the mouse hypothalamus. As a mechanism, it is speculated that abundant ATP supply reinforces the UPR, which requires appreciable amounts of ATP. However, the precise mechanism by which ATP ameliorates ER stress is also unclear.

Previous studies demonstrated that overexpression of X-box binding protein 1 (XBP1) in in vitro and in vivo settings reduces ER stress and increases leptin sensitivity^10,33^. It was also reported that chemical chaperones such as 4-phenyl butyrate (4-PBA) and tauroursodeoxycholic acid (TUDCA), which are agents that decrease ER stress, increase leptin sensitivity^10,34^. Because treatment of 4-PBA reduces ER stress even in the absence of XBP1^34^, each manipulation regulates ER stress via independent pathways. Celastrol, which was a small molecule discovered by analyzing the effects of both XBP1 and 4-PBA, was also demonstrated to decrease ER stress and increase leptin sensitivity in the hypothalamus^35^. The effect of Celastrol on body weight is more potent than 4-PBA or TUDCA^35^. ER stress response has been shown to have multiple pathways^36^. Although the present study demonstrated that BD treatment decreases ER stress in the hypothalamus, the details of pathways remains unclear. More elaborate studies are needed to elucidate the pathway by which ketone bodies or ATP decreases ER stress.

In conclusion, this is the first report demonstrating the leptin sensitizing effect of BD treatment in DIO mice with leptin resistance. BD treatment effectively increased plasma βHB and hypothalamic ATP concentrations and ameliorated ER stress and leptin resistance at the hypothalamic level. The present study suggests the possibility that the effect of BD treatment is mediated by the elevation of hypothalamic ATP concentration. Ketone bodies and hypothalamic ATP are the potential targets for the treatment of obesity and its complications.

## Methods

### Materials

All reagents were purchased from Sigma-Aldrich (St Louis, MO) unless otherwise mentioned.

### Animals

Male C57BL/6J wild type and *ob/ob* mice were purchased from Japan SLC (Shizuoka, Japan). Mice were housed under conditions of controlled temperature (23 °C ± 1 °C), humidity (55% ± 5%) and lighting (light phase 7:30–19:30) and fed an ad libitum standard diet (SD) (MF; Oriental Yeast, Tokyo, Japan). For high fat diet (HFD) loading, mice were fed an ad libitum HFD containing 20% kcal for kcal (kcal/kcal) protein, 20% kcal/kcal carbohydrate, and 60% kcal/kcal fat (D12492; Research Diets, New Brunswick, NJ) from the ages of 8 weeks. To induce DIO in mice, HFD loading was conducted for 8 weeks. All animal experiments were carried out after receiving approval from the Institutional Animal Experiment Committee and in accordance with the Institutional Regulation for Animal Experiments at Jichi Medical University.

### BD treatment

BD was administered twice daily by oral gavage at the dose of 5 g/kg at 8:00 and 17:00. As a control, water of the same volume was administered. The calorie of BD used for treatment was added to the amount of food intake for BD treatment groups.

### Co-administration experiment of leptin and BD

Leptin (Amylin Pharmaceuticals, San Diego, CA) was intraperitoneally injected at the dose of 150 μg/kg at 17:00. As a control, saline of the same volume was injected. BD was administered as described above. For an experiment with *ob/ob* mice, 12 weeks old mice were divided into three treatment groups of ‘saline + water’, ‘leptin + water’ and ‘leptin + BD’ to be counterbalanced for starting body weight. For an experiment under HFD, 8 weeks old wild type mice were divided into four treatment groups of ‘saline + water’, ‘leptin + water’, ‘saline + BD’ and ‘leptin + BD’ to be counterbalanced for starting body weight. HFD feeding was continued during the experiment.

### Pair-feeding experiment

Pair-feeding experiment was performed under HFD. Eight weeks old wild type mice were divided into three groups of ‘water’, ‘BD’ and ‘water + pair-fed’ to BD administered mice not to make differences in starting body weight among groups. For pair-fed mice, food of the same amount consumed by BD administered mice during light and dark phases on the previous day was placed at 8:00 and 17:00, respectively.

### Biochemical assays

Blood was obtained from the retro-orbital sinus after 4 h fasting. Plasma leptin concentrations were measured by ELISA kit for rat leptin (Millipore, St. Charles, MO). Plasma glucose concentrations were measured by a glucose assay kit (Wako Pure Chemical Industries, Osaka, Japan). Plasma insulin concentrations were measured by an insulin-ELISA kit (Morinaga Institute of Biological Science, Yokohama, Japan). Plasma triglyceride (TG), nonesterified fatty acid (NEFA), and total cholesterol concentrations were measured by enzymatic kits (Triglyceride E-test Wako, NEFA C-test Wako, and Cholesterol E-test Wako, respectively; Wako Pure Chemical Industries). For measurement of plasma βHB concentrations, blood was obtained 4 h after water or BD administration. Plasma βHB concentrations were measured by beta Hydroxybutyrate Assay Kit (abcam, Cambridge, MA).

### Calculation of HOMA of insulin resistance

The HOMA of insulin resistance (HOMA-IR) was calculated as an indicator of insulin sensitivity according to Eq. (1):

1 $$ {\text{insulin }}\left( {\upmu {\text{IU}}/{\text{mL}}} \right) \times {\text{glucose }}\left( {{\text{mg}}/{\text{dL}}} \right)/405 $$

### Measurement of hypothalamic ATP concentrations

Whole hypothalami were obtained from mice 4 h after water or BD administration. ATP levels were measured by using the luciferase-based Tissue ATP assay Kit (TOYO B-Net, Tokyo, Japan). As described in the instruction, whole hypothalami were lysed in lysis buffer. After centrifugation (4 °C 1000×*g*, 10 min), the supernatant was incubated with ATP extraction regent at room temperature for 30 min. The samples (100 μl) mixed with 100 μl of ATP luminescence reagent were measured by luminometer GL-200 (MICROTEC, Funabashi, Japan) and calculated.

### Measurement of rectal temperature

The rectal temperature was measured by a digital thermometer (BAT-7001H, Physitemp Instruments, Inc., Clifton, NJ) in animal rooms at 9:00.

### Assessment of leptin sensitivity

Water or BD (5 g/kg) was administered at 8:00 and 17:00 twice daily for three days to 16-week-old lean or DIO mice in which had been fed HFD for 8 weeks. Water and BD groups were divided into two sub-groups and mice were intraperitoneally injected either with saline or leptin (150 μg/kg) at 10:00 on the third day. Food intake were monitored for 24 h after saline or leptin injection. For western blot analysis for phosphorylation of STAT3, whole hypothalami were obtained from mice 1 h after saline or leptin injection.

### Western blot analysis

Whole hypothalami were homogenized and lysed in a solution containing 20 mM Tris (pH 7.5) 150 mM NaCl, 1 mM EDTA, 1 mM EGTA, 1% Triton X-100, 2.5 mM sodium pyrophosphate and 1 mM sodium orthovanadate; the lysates were analyzed for protein content before SDS-PAGE analysis. For the analysis of ER stress markers, whole hypothalamic were obtained from mice on the last day of each experiment. Membranes were immunoblotted with each antibody to phospho-STAT3 (Tyr705), STAT3, CHOP, β-actin, phospho-PERK (Thr980) and PERK. All antibodies were purchased from Cell Signaling Technology (Beverley, MA). Amersham ECL prime (GE Healthcare Life Sciences, Pittsburgh, PA) and ImageQuant LAS 4000mini (GE Healthcare Life Sciences) were used for the detection.

### Statistics

Data are expressed as means ± SEM. Comparison between or among groups was assessed by Student’s *t* test or Bonferroni's multiple comparison test. *P* < 0.05 was considered statistically significant.

## Supplementary Information

Supplementary Figures.
