# Supplementary material for: Leptin sensitizing effect of 1,3-butanediol and its potential mechanism
Source: Sci Rep. 2021 Sep 6;11:17691. doi: 10.1038/s41598-021-96460-y (PMC8421515; doi:10.1038/s41598-021-96460-y)
Supplement: Supplementary file 1 — Supplementary Figures. [file 41598_2021_96460_MOESM1_ESM.pptx]

## Slide 1
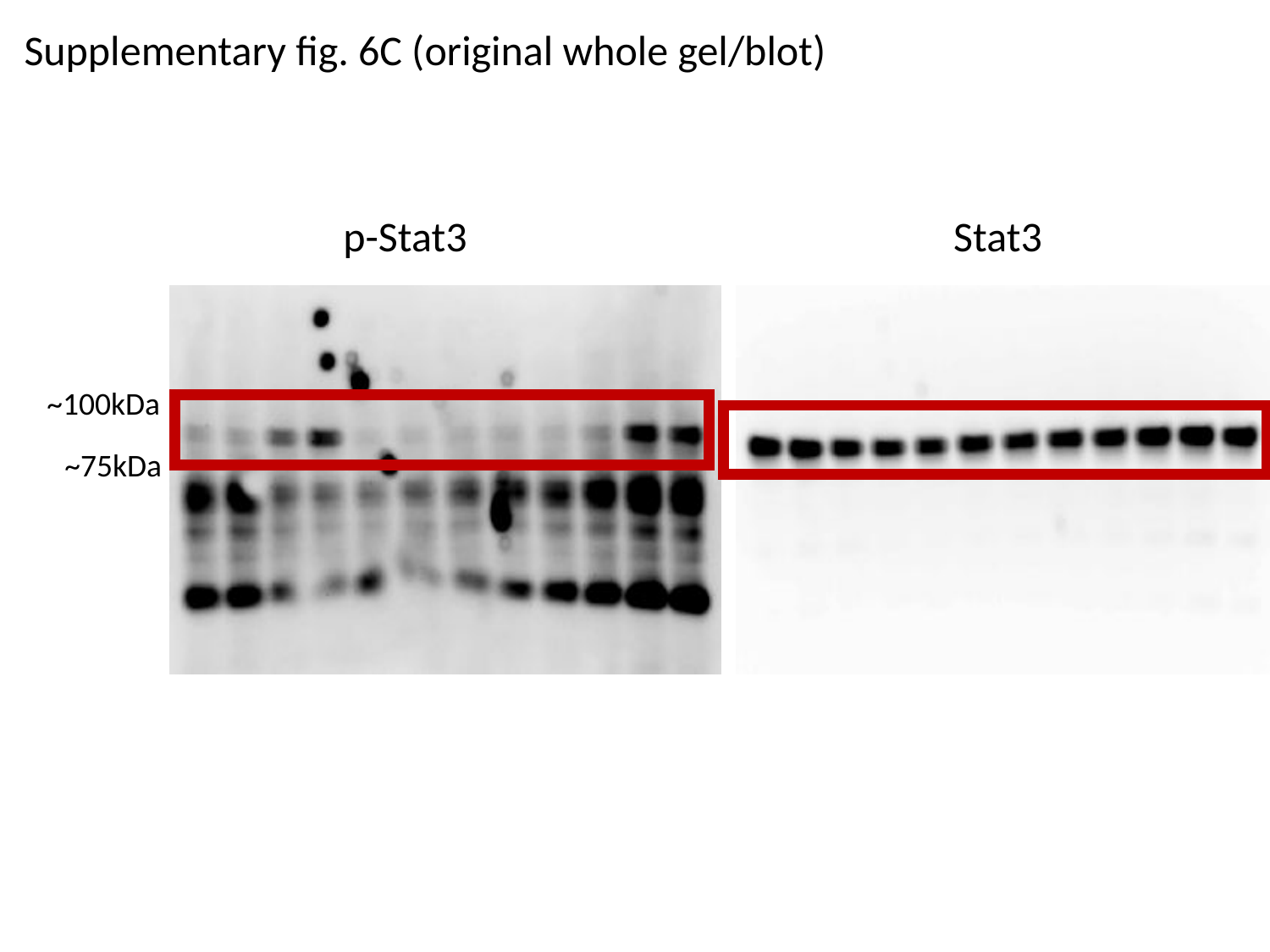

Supplementary fig. 6C (original whole gel/blot)
p-Stat3
Stat3
~100kDa
~75kDa

## Slide 2
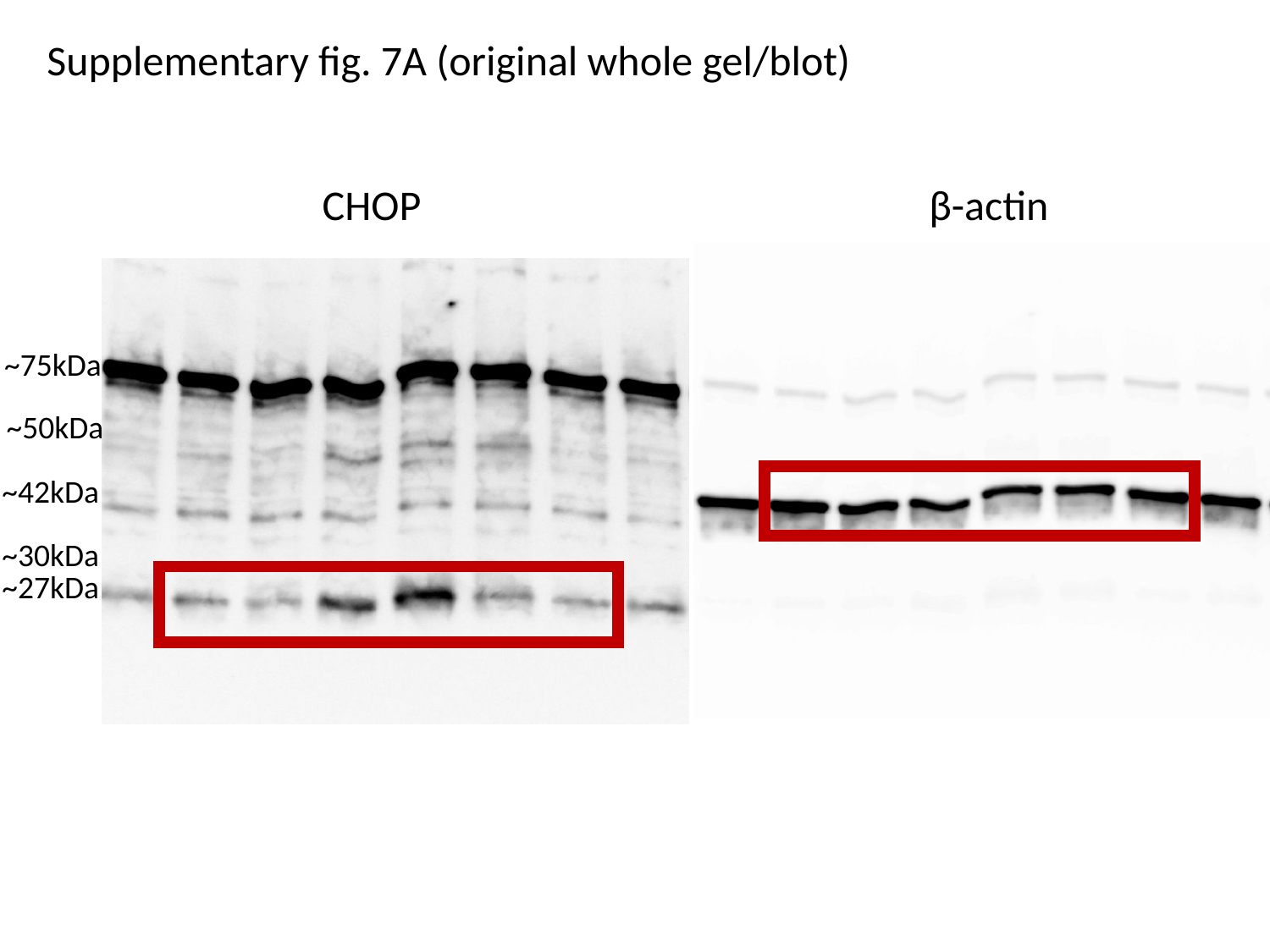

Supplementary fig. 7A (original whole gel/blot)
CHOP
β-actin
~75kDa
~50kDa
~42kDa
~30kDa
~27kDa

## Slide 3
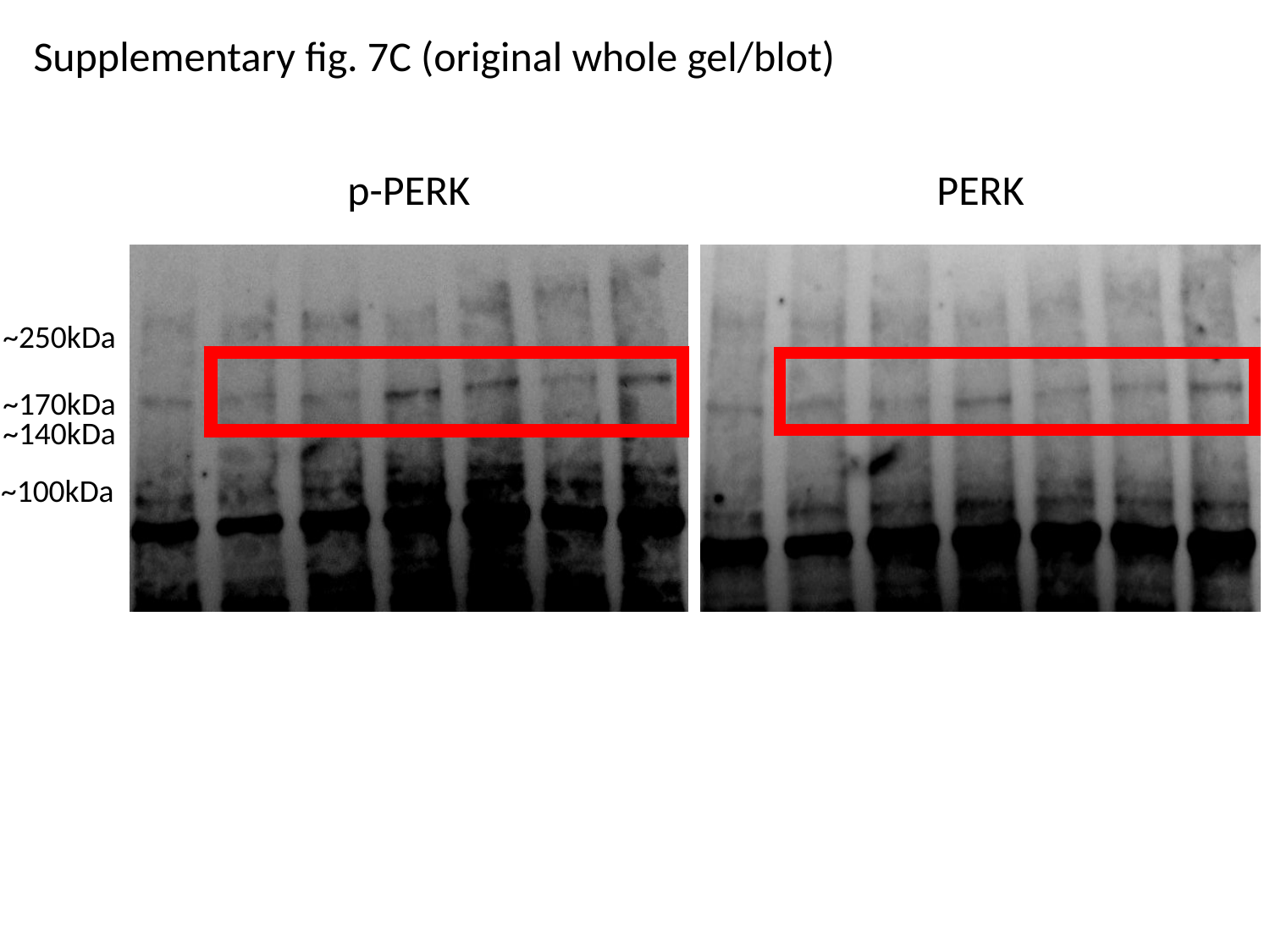

Supplementary fig. 7C (original whole gel/blot)
PERK
p-PERK
~250kDa
~170kDa
~140kDa
~100kDa

## Slide 4
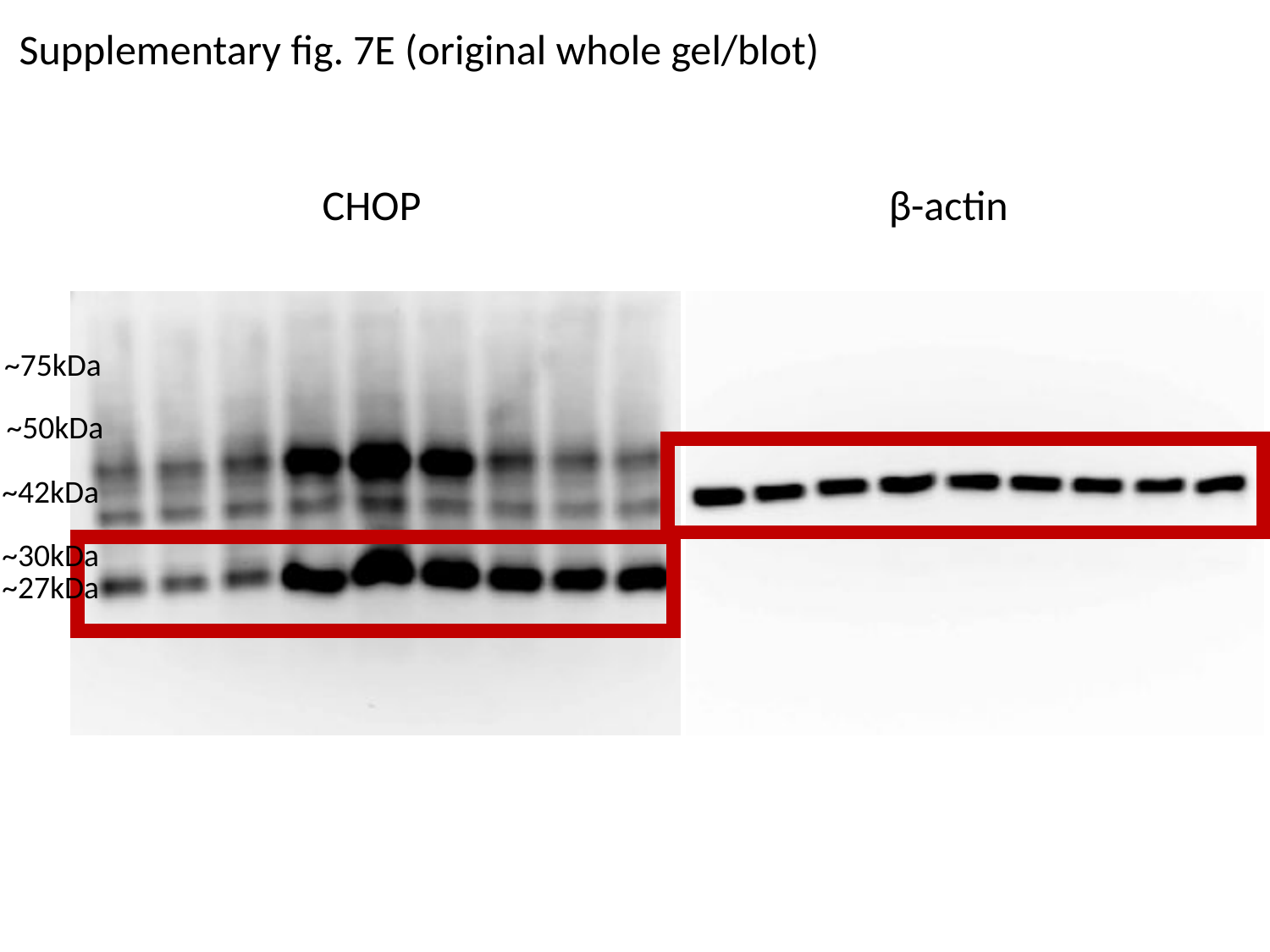

Supplementary fig. 7E (original whole gel/blot)
β-actin
CHOP
~75kDa
~50kDa
~42kDa
~30kDa
~27kDa

## Slide 5
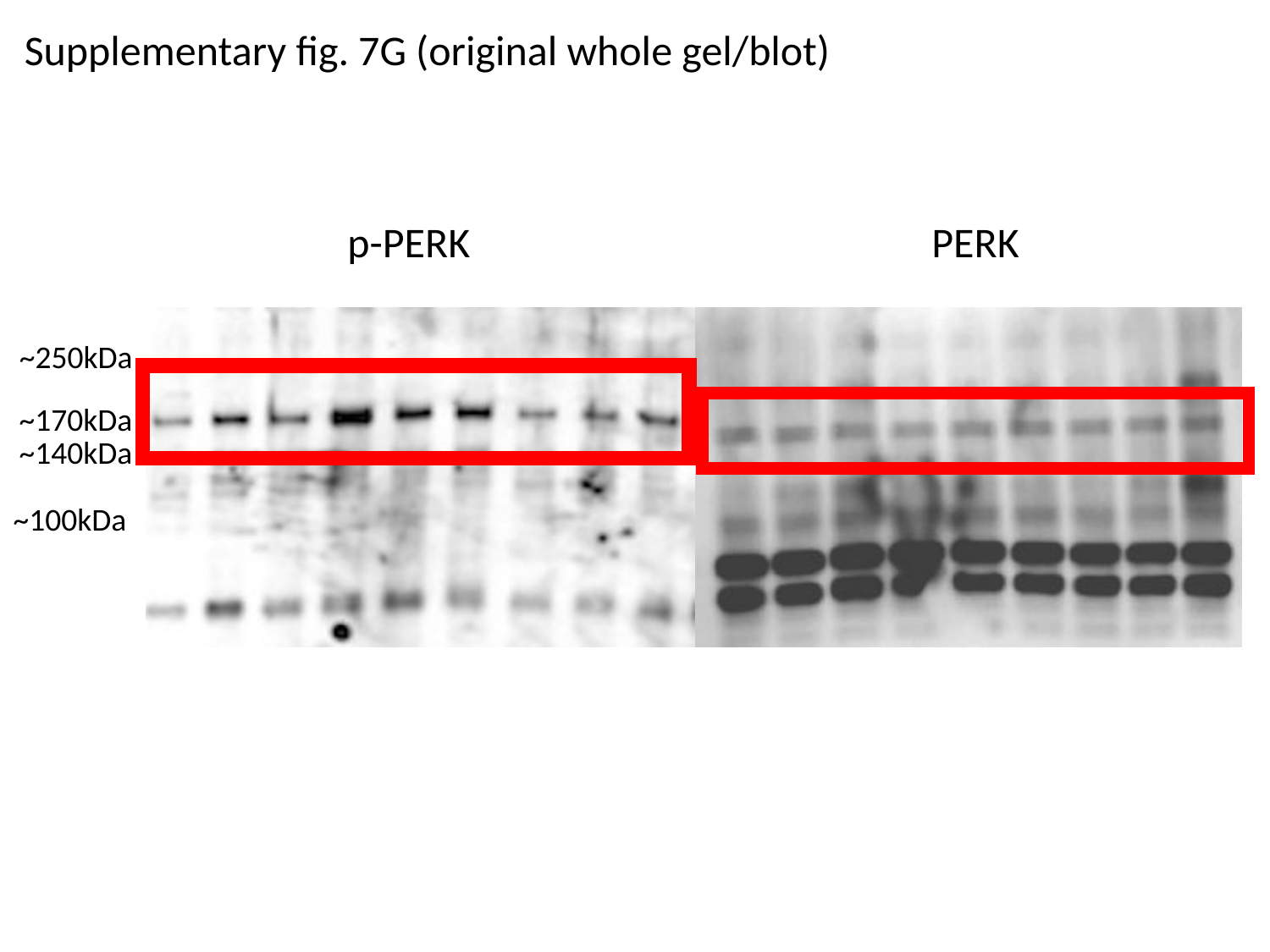

Supplementary fig. 7G (original whole gel/blot)
p-PERK
PERK
~250kDa
~170kDa
~140kDa
~100kDa
